# Seronegative Paraneoplastic Opsoclonus–Myoclonus–Ataxia Syndrome Secondary to Low Volume Endocrine-Sensitive Malignancy of Likely Breast Origin

**DOI:** 10.3390/curroncol32080440

**Published:** 2025-08-06

**Authors:** Geraint Berger, Caitlin Jackson-Tarlton, Daniel Rayson, Alexander Silver, Mark Walsh, Ashley Drohan

**Affiliations:** 1 Faculty of Medicine, Dalhousie University, Halifax, NS B3H 4R2, Canada; gcberger@dal.ca (G.B.); caitlin.tarlton@nshealth.ca (C.J.-T.); daniel.rayson@nshealth.ca (D.R.); al478160@dal.ca (A.S.); mark.walsh@nshealth.ca (M.W.); 2 Department of Surgery, Division of General and Gastrointestinal Surgery, Dalhousie University, Halifax, NS B3H 2Y9, Canada; 3 Department of Medicine, Division of Neurology, Dalhousie University, Halifax, NS B3H 3A7, Canada; 4 Department of Medicine, Division of Medical Oncology, Dalhousie University, Halifax, NS B3H 2Y9, Canada

**Keywords:** opsoclonus, myoclonus, ataxia, paraneoplastic, malignancy, poorly differentiated carcinoma

## Abstract

A 51-year-old female presented to the emergency department with vertigo, visual disturbances, involuntary rapid repetitive eye movements, incoordination, and imbalance. Physical examination revealed opsoclonus, myoclonus, and bilateral limb and gait ataxia. Initial workup was negative for intracranial abnormalities, and no abnormalities were noted on blood work or cerebrospinal fluid analysis. Tumor markers were within normal limits. As part of her diagnostic workup, a positron emission tomography (PET) scan was performed, which showed a highly FDG-avid solitary 7 mm left axillary lymph node. Ultrasound-guided percutaneous biopsy revealed metastatic poorly differentiated carcinoma. Histopathological examination could not conclusively distinguish between adenocarcinoma and squamous cell carcinoma. She was diagnosed with seronegative opsoclonus-myoclonus ataxia syndrome of paraneoplastic origin from an occult primary malignancy and started on pulsatile corticosteroids and intravenous immunoglobulin (IVIG), with only moderate symptomatic improvement. Given the anatomic location and immunohistochemical staining pattern of the lymph node, the malignancy was considered as being of primary breast origin. A left axillary lymph node dissection was performed, with 1/12 nodes testing positive for poorly differentiated carcinoma. The patient experienced significant improvement in her neurological symptoms 2–3 days following resection of the solitary malignant lymph node, largely regaining her functional independence. She went on to receive adjuvant radiotherapy to the breast and axilla, as well as adjuvant hormonal therapy.

## Simple Summary

This case report details a patient who presented to the emergency department on various occasions with several debilitating neurological symptoms of unexplained etiology. Several treatments were trialed but did not provide lasting benefit. As part of a detailed work-up, a PET scan was performed, which revealed a highly suspicious lymph node in the axilla. Subsequent biopsy of this lymph node revealed cancer; however, the primary site remained indeterminate following additional imaging. The combination of the patient’s unique neurological presentation in the setting of malignancy increased the suspicion of a rare paraneoplastic syndrome, termed opsoclonus–myoclonus–ataxia syndrome. Various immunosuppressive treatments were initiated and provided moderate benefit. Surgical removal of the axillary lymph node, however, resulted in profound improvement in the patient’s condition.

## 1. Introduction

Paraneoplastic syndromes are a group of disorders associated with a variety of malignancies that are not directly attributable to metastatic invasion, tumor compression, metabolic abnormalities, infections, malignancy-associated coagulopathies, or side effects from treatment of the primary neoplasm [1]. They are a heterogeneous group of disorders that are caused by tumor secretion of functional peptides, hormones, or cytokines (paraneoplastic endocrine syndromes, PESs), or by immune-mediated cross reactivity between malignant and normal tissues (paraneoplastic neurologic syndromes, PNSs) [2]. In the case of PES, the bioactive molecules produced by the malignant cells lead to metabolic derangements. Examples include syndrome of inappropriate antidiuretic hormone secretion (SIADH), hypercalcemia of malignancy, and ectopic ACTH leading to Cushing syndrome [3]. While some paraneoplastic neurologic syndromes can be caused by a similar mechanism, the majority are immune-mediated. These syndromes are triggered by an immune response against onconeural antigens that are expressed by the patients’ tumor, as well as by neurons within the nervous system (cross-reactivity) [4]. Due to the antigenic similarity, the onconeural antibodies and associated T lymphocytes target different components of the nervous system [2]. Paraneoplastic neurologic syndromes can affect both central and peripheral nervous systems and/or neuromuscular junctions, with symptoms resulting from the affected neuronal elements [5]. Paraneoplastic opsoclonus–myoclonus–ataxia (OMS) syndrome is a rare, debilitating PNS characterized by acute or subacute onset of spontaneous high-frequency involuntary saccadic eye movements (opsoclonus), with spontaneous non-epileptic myoclonus [6]. It can be accompanied by cerebellar ataxia, aphasia, mutism, and sleep disturbance [6]. Etiologies of OMS include paraneoplastic, parainfectious, toxic-metabolic, and idiopathic [6]. The presence of the onconeural auto-antibodies anti-Hu and anti-Ri is most strongly associated with this syndrome; however, many OMS patients are seronegative for all known antineural antibodies [7]. Here we present the case of a patient presenting with seronegative OMS in the setting of a malignancy of indeterminate primary origin.

## 2. Case Description

A 51-year-old female initially presented to the emergency department (ED) in early July of 2023 with an acute onset of severe vertigo. She had four visits to the emergency department over 9 days, the time course of which is presented below.

Past medical history was negative for prior neurological or systemic conditions. There was no personal or family history of malignancy. The patient was diagnosed with a viral gastroenteritis 2 weeks prior to presentation and had mild, intermittent upper abdominal pain along with a 5-day history of vasomotor symptoms, night sweats, nausea, vomiting, and a sensation of tremulousness. There was no history of smoking, intravenous drug use, or alcohol consumption. Her only medication was hormone replacement therapy, recently started by her family physician for symptoms of perimenopause. An Epley maneuver was felt to provide some relief. Suspected etiologies for her symptoms included the recent GI illness, perimenopause, and/or the peripheral vertiginous syndrome.

Two days later, she re-presented to the ED with worsening symptoms, including tinnitus in the right ear and involuntary brief jerking movements of her arms, trunk, and legs. There was no interval history of head trauma. Cranial nerve examination was unremarkable, as was blood work, and she was started on a short course of prednisone for suspected post-viral peripheral vestibulopathy, but re-presented 3 days later via ambulance with worsening symptoms. Computed tomography (CT) of the head showed no intracranial abnormalities. She was diagnosed with suspected labyrinthitis and started on cefuroxime. Two days later, she returned tachycardic and tremulous with an unsteady gait. At this time, Neurology was consulted. Neurological exam revealed opsoclonus (Supplementary Materials Video S1) in addition to dysarthria and a tremulous voice. There was no peripheral pattern of nystagmus, and the cranial nerve exam was unremarkable. She had paratonia, but no true increase in tone, and strength was full throughout. She had diffuse symmetric grade 3 hyperreflexia throughout with equivocal plantar reflexes. She also had intermittent myoclonic jerks of her head, trunk, arms, and legs. Tests of coordination with finger-to-nose, rapid alternating hand movements, and heel-to-shin showed dysmetria, dysdiadochokinesia, and bilateral ataxia. The sensory exam was within normal limits. Her gait was wide-based and unsteady, which was further impaired by her eye movements and myoclonic jerks. She was admitted to General Neurology with a working diagnosis of opsoclonus myoclonus ataxia syndrome, with infectious, post-infectious, and paraneoplastic as possible causes. She was started empirically on intravenous immunoglobulin (IVIG, 2 g/kg daily for 5 days) as an immune etiology was highly suspected.

**Figure 1:**
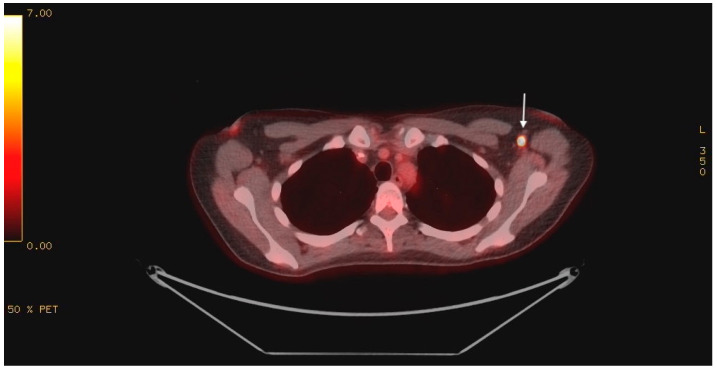
PET scan showing intense 18-FDG uptake in the left axilla (indicated by white arrow).

Magnetic resonance imaging (MRI) of the brain with gadolinium showed no evidence of acute cerebral ischemia, intracranial hemorrhage, encephalitis (including cerebellitis), mass, or evidence of leptomeningeal disease. Cerebrospinal fluid (CSF) analysis showed a normal cell count, protein, glucose, negative testing for a comprehensive viral PCR panel, negative bacterial cultures, and no evidence of malignant cells on cytology. Given the MRI brain and CSF findings, infectious and post-infectious etiologies became less likely, and a paraneoplastic investigation ensued. Paraneoplastic panel in serum was negative for the following autoantibodies: myositis-related antigen, Hu (ANNA-1), Yo, Ri (NOVA-1), PNMA2 (Ma2Ta), Ampiphysin, CV2.1, Recoverin, SOX1, Titin, GAD65, Zic4, and Tr. Tumor markers, including CEA, CA19-9, AFP, beta-hCG, CA-125, and CA15-3, were all within normal limits. CTs of the chest, abdomen, and pelvis were unremarkable. Pelvic ultrasound ruled out a gynecologic malignancy. The patient underwent a positron emission tomography (PET) scan, which revealed intense 18-FDG uptake in a solitary, 7 mm left axillary lymph node with a maximum standard unit value (SUV Max) of 19.0 (Figure 1). No other findings of concern were identified.

**Figure 2:**
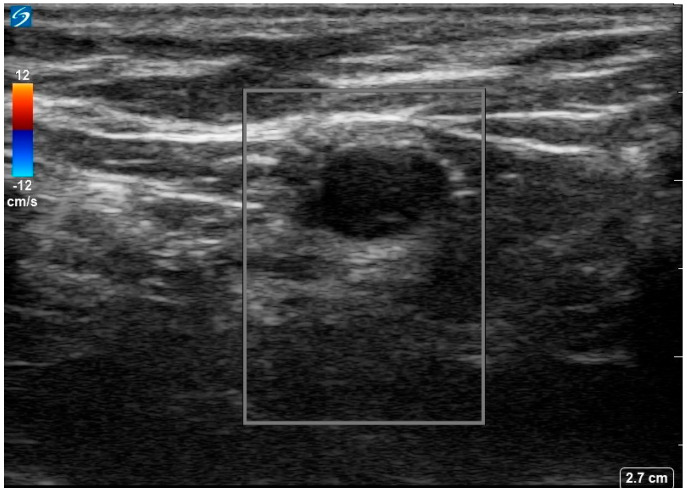
Ultrasound image of the 18−FDG avid left axillary lymph node. Image shows a 0.9 × 0.6 × 0.7 cm markedly hypoechoic lesion with loss of normal fatty hilum, reniform shape, and irregular margins with prominent peripheral feeding vessels.

She was diagnosed with seronegative opsoclonus-myoclonus ataxia syndrome of paraneoplastic origin. Given suboptimal response to IVIG, she was started on 1 g IV methylprednisolone for 5 days. An ultrasound of the left axilla identified a 0.9 × 0.6 cm hypoechoic node with notable loss of the normal fatty hilum and reniform shape, irregular margins and prominent peripheral feeding vessels (Figure 2.

**Figure 3:**
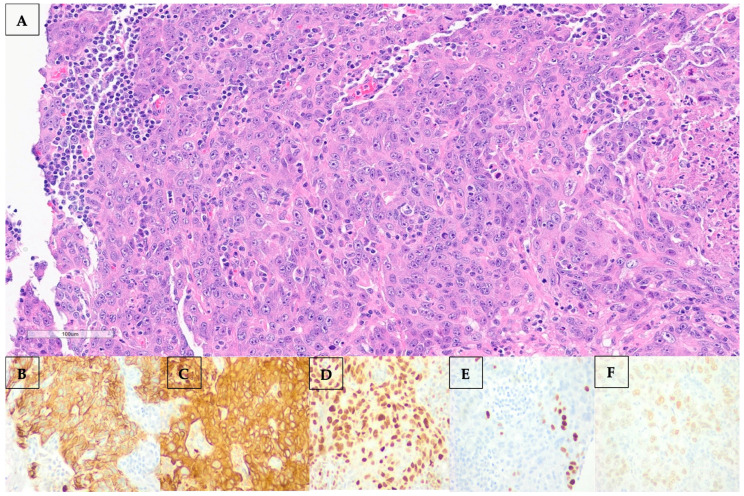
(**A**–**F**): (**A**) Hematoxylin and eosin (H&E) staining of the left axillary lymph node showing marked atypia, focally quite pick cytoplasm, focal necrosis, and mitotic figures. By immunostaining, the neoplasm stained positive for CK7 (**B**), AE1AE3 (**C**), diffusely positive for GATA3 (**D**), focally positive for p40 (**E**), and 20% positive for estrogen receptor (ER) (**F**).

Percutaneous biopsy of the axillary node revealed a metastatic poorly differentiated carcinoma. Morphological examination and immunostaining could not conclusively distinguish between adenocarcinoma and squamous cell carcinoma. Histological and immunohistochemical images are shown in Figure 3. Of note, the neoplasm was negative for the following: CK20, TTF-1, Napsin, Synaptophysin, Chromogranin, S100, HMB45, Mart-1, Mammaglobin, GCDFP, ER, PR, HER2, CDX2, PAX8, and CD45.

Mammography and bilateral gadolinium-enhanced breast MRI were both performed. The MRI showed only benign-appearing foci of gadolinium uptake in both breasts. A left axillary lymph node dissection was performed, with 1/12 lymph nodes found to be positive for poorly differentiated carcinoma. The patient reported rapid, dramatic improvement in her neurological symptoms 2-3 days following resection of the solitary lymph node. She underwent adjuvant radiotherapy to the breast and axilla and was also started on tamoxifen 20 mg orally daily. She was maintained on a prolonged course of prednisone, which was tapered and stopped approximately 13 months after surgery. Prior to surgical resection, she was non-ambulatory and wheelchair dependent due to the opsoclonus, myoclonic jerks, and ataxia. She required assistance for the majority of her activities of daily living (ADLs) and instrumental ADLs (IADLs). Within days after surgery, she had regained her ability to ambulate and was largely functionally independent. At her most recent follow-up, 20 months post-axillary node dissection, she was able to participate in hiking, skating, and biking, and was able to read, with some residual symptoms, including fatigue and occasional imbalance.

## 3. Discussion

While considered rare, affecting an estimated 1 in 300 cancer patients, certain malignancies carry a greater risk for the development of paraneoplastic neurological syndromes [8]. Up to 5% of patients diagnosed with small cell lung cancer and 10% of patients with lymphoma develop some form of PNS [9]. The most common manifestations of PNS include limbic encephalitis, cerebellar degeneration, and encephalomyelitis, with OMS representing approximately 1% of all PNS cases [8]. OMS is most commonly seen in association with pediatric neuroblastoma, with an estimated 50% of pediatric patients presenting with OMS having an underlying neuroblastoma [10]. Cases of paraneoplastic OMS have been reported in association with breast cancer [11], melanoma [12], lung cancer [13], and urogenital cancers [14].

In many instances, the neurological symptoms of PNS antedate the diagnosis of the malignancy, which can be occult [6]. Medical management remains largely based on expert opinions, with most recommendations stressing the importance of detection and treatment of the underlying malignancy to optimize chances of symptom control [10]. Aside from specific oncological treatment, first-line therapy for paraneoplastic OMS consists of corticosteroids, intravenous immunoglobulins, plasmapheresis, and rituximab [6,15]. One of the only randomized trials investigating OMS treatment demonstrated that for children with neuroblastoma-related OMS treated with prednisolone, with or without IVIG, along with risk-adapted chemotherapy, a significantly higher OMS response rate was observed, favoring the addition of IVIG [16]. In cases of steroid-refractory OMS, plasmapheresis has been shown to yield positive results [17].

In this case report, symptom control was achieved following surgical resection of the malignant lymph node. Although the underlying autoimmune process is initiated in the tumor microenvironment, OMS symptoms can persist following effective or curative treatment of the underlying malignancy [11]. This highlights the need for multidisciplinary involvement of neurology, surgical, and oncology teams with concurrent immunosuppressive therapy and antineoplastic intervention, along with close monitoring of symptoms when it is feasible, as it was in our case. Immunosuppressive therapy should continue based on clinical response and ongoing symptoms with close neurological follow-up care.

Due to the rarity of paraneoplastic OMS, longer-term outcome data are limited; however, significant clinical improvement and eventual full recovery have been reported [18]. In the presented case, the patient experienced rapid, dramatic improvement following surgical resection of the malignant lymph node and with tapered corticosteroid therapy. She regained her functional independence post-operatively, whereas she was wheelchair-dependent immediately prior to surgery and required assistance for her ADLs and IADLs. Given the rarity of paraneoplastic OMS, a high degree of suspicion is required to diagnose this often-debilitating condition. Early recognition, accurate diagnosis, and rapid treatment are of utmost importance to optimize chances of a favorable neurological and oncologic outcome.

## Data Availability

No new data were created or analyzed in this study. Data sharing is not applicable to this article.

## Supplementary Materials

The following supporting information can be downloaded at: https://www.mdpi.com/article/10.3390/curroncol32080440/s1. Video S1: Video demonstrating the patient’s opsoclonus on physical examination (rapid, involuntary, arrhythmic, and multidirectional eye movements).
